# The Swiss Pathogen Surveillance Platform – towards a nation-wide One Health data exchange platform for bacterial, viral and fungal genomics and associated metadata

**DOI:** 10.1099/mgen.0.001001

**Published:** 2023-05-12

**Authors:** Aitana Neves, Daniel Walther, Trinidad Martin-Campos, Valerie Barbie, Claire Bertelli, Dominique Blanc, Gérard Bouchet, Frédéric Erard, Gilbert Greub, Hans H. Hirsch, Michael Huber, Laurent Kaiser, Stephen L. Leib, Karoline Leuzinger, Vladimir Lazarevic, Mirjam Mäusezahl, Jorge Molina, Richard A. Neher, Vincent Perreten, Alban Ramette, Tim Roloff, Jacques Schrenzel, Helena M. B. Seth-Smith, Roger Stephan, Dillenn Terumalai, Fanny Wegner, Adrian Egli

**Affiliations:** ^1^​ SIB Swiss Institute of Bioinformatics, Geneva, Switzerland; ^2^​ Clinical Microbiology, University Hospital Lausanne, Lausanne, Switzerland; ^3^​ Hospital Epidemiology, University Hospital Lausanne, Lausanne, Switzerland; ^4^​ Clinical Virology, University Hospital Basel, Basel, Switzerland; ^5^​ Department of Biomedicine, Transplantation & Clinical Virology, University of Basel, Basel, Switzerland; ^6^​ Institute of Medical Virology, University of Zurich, Zurich, Switzerland; ^7^​ Virology, University Hospital Geneva, Geneva, Switzerland; ^8^​ Institute for Infectious Diseases (IFIK), University of Bern, Bern, Switzerland; ^9^​ Multidisciplinary Center for Infectious Diseases, University of Bern, Bern, Switzerland; ^10^​ Genomic Research Laboratory, University of Geneva, Geneva, Switzerland; ^11^​ Federal Office of Public Health, Bern, Switzerland; ^12^​ Biozentrum, University of Basel, Basel, Switzerland; ^13^​ SIB Swiss Institute of Bioinformatics, Basel, Switzerland; ^14^​ Institute of Veterinary Bacteriology, Vetsuisse Faculty, University of Bern, Bern, Switzerland; ^15^​ Institute of Medical Microbiology, University of Zurich, Zurich, Switzerland; ^16^​ Institute for Food Safety and Hygiene, Vetsuisse Faculty, University of Zurich, Zurich, Switzerland

**Keywords:** molecular surveillance, research, sequencing, bacteria, virus, fungi, antibiotic resistance, virulence, outbreaks, typing, epidemiology, database, bioinformatics

## Abstract

The Swiss Pathogen Surveillance Platform (SPSP) is a shared secure surveillance platform between human and veterinary medicine, to also include environmental and foodborne isolates. It enables rapid and detailed transmission monitoring and outbreak surveillance of pathogens using whole genome sequencing data and associated metadata. It features controlled data access, complex dynamic queries, dedicated dashboards and automated data sharing with international repositories, providing actionable results for public health and the vision to improve societal well-being and health.

## Data Summary

The SARS-CoV-2 raw datasets and consensus genomes published by the Swiss Pathogen Surveillance Platform can be found on the EU Covid-19 Data Portal page: https://www.covid19dataportal.org.

The nextflow pipelines developed within the platform are available on a Gitlab repository (currently under development): https://gitlab.sib.swiss/clinbio/spsp-ng/spsp-ng-bioinformatics.

The code of the platform will be released soon under the Gitlab repository of the developing team: https://gitlab.sib.swiss/clinbio/spsp-ng.

**Impact Statement**

This article provides a practical use case and introduction on how to build and maintain a national surveillance database infrastructure for sharing whole genome sequencing data and associated epidemiological metadata. The Swiss Pathogen Surveillance Platform has been built as a One Health-focused platform and was used during the COVID19 pandemic. The built framework may serve as a template to build future platforms.

## Introduction

Whole genome sequencing (WGS) of bacterial, viral and fungal isolates is the preferred typing method and has become a reference standard due to its high resolution [1, 2]. Various publications and studies have shown the improved resolution and the potential for high throughput in comparison with techniques such as PFGE and multi-locus sequencing typing [3–5]. The analysis of microbial sequencing data allows the assessment of the genomic relatedness during outbreak investigations and source tracking, as well as the identification of microbial species, antimicrobial resistance or virulence genes based on genomic content. The transmission of individual strains can be monitored across and among different human, animal and environmental sources – a concept also known as One Health [6]. The analysis of sequencing data requires bioinformatic tools to assemble, annotate and compare the genomes [7]. During the past 2 years of the SARS-CoV-2 (severe acute respiratory syndrome coronavirus 2) pandemic, tremendous sequencing capacities for surveillance have been built in many institutions or at national levels, which have provided very rapid insights into the transmission dynamics and evolution of SARS-CoV-2 [8]. Globally, more than 14.8 million SARS-CoV-2 genomes have been sequenced and data are usually accessible in public data repositories such as the European COVID-19 Data Portal [9] and GISAID [10], as well as processed and visualized with platforms such as nextstrain [11]. Over the past 5–10 years, genomes of isolates from many bacterial, viral and fungal species have been sequenced and deposited in databases, due to the lower sequencing costs and massively increased sequencing capacities and demands. This trend is unlikely to stop soon [12]. These data are often re-used in diverse national or even global surveillance systems, such as for foodborne pathogens [2].

Despite the large potential of WGS for surveillance and research, several challenges remain. Important open questions in the field are: (a) How can we generate sustainable surveillance for critical pathogens following a One Health concept? (b) What represents enough isolates sequenced for respiratory and non-respiratory pathogens? (c) Can we set up a surveillance system which would prepare us for future pandemic events? To implement a sustainable data platform we must address critical factors such as: (a) the collection, storage, access and sharing of sequencing data and associated epidemiological metadata in standardized, interoperable formats, improving overall data quality; (b) the ethical and legal framework, as well as technical implementation of sensitive metadata access for in-depth surveillance including spatio-temporal monitoring for different stakeholders; and (c) the implementation of standards and harmonization in the pre- to post-analytical processes for research and surveillance. The Swiss Pathogen Surveillance Platform (SPSP.ch) was built with the vision to enable rapid and detailed transmission monitoring and outbreak surveillance of pathogens [13]. In this article we introduce SPSP and describe its core technical structure, its governance setup and a roadmap for future developments.

### Building and establishing SPSP

To address these challenges, we developed a prototype of SPSP from 2018 to 2020 based on methicillin-resistant *Staphylococcus aureus* thanks to an antimicrobial resistance funding programme. This initiative was driven by the Swiss clinical and veterinary communities and was innovative in that it enabled fine-grained controlled data access and sharing of clinical genomic data together with potentially sensitive personal data, within an ethically and legally controlled framework. While other platforms existed at the time to share genomic data and visualize phylogenetic trees, such as nextstrain.org and IRIDA.ca, they did not allow for sufficiently granular user management and definition of data access levels at the level of metadata for our envisaged use cases, such as sharing minimal metadata on samples with all users, and sensitive metadata on these samples with only public health authorities or a subset of users. Early in 2021, SPSP was mandated by the State Secretariat for Education, Research and Innovation (SERI) and the Federal Office of Public Health (FOPH) to act as the national central data hub for sharing SARS-CoV-2 genomic data with the European COVID-19 Data Portal and GISAID. As of October 2022, SPSP had collected more than 140 000 SARS-CoV-2 genomes.

### A well-defined governance and ethical/legal framework

SPSP brings together all major stakeholders and associated experts in Swiss healthcare and public health, including human and veterinary microbiology laboratories, infectious disease and hospital epidemiology, cantonal physicians and laboratories, the FOPH, the Federal Food Safety and Veterinary Office (FSVO), as well as several central laboratories with reference functions and also regional and private laboratories.

**Fig. 1.**
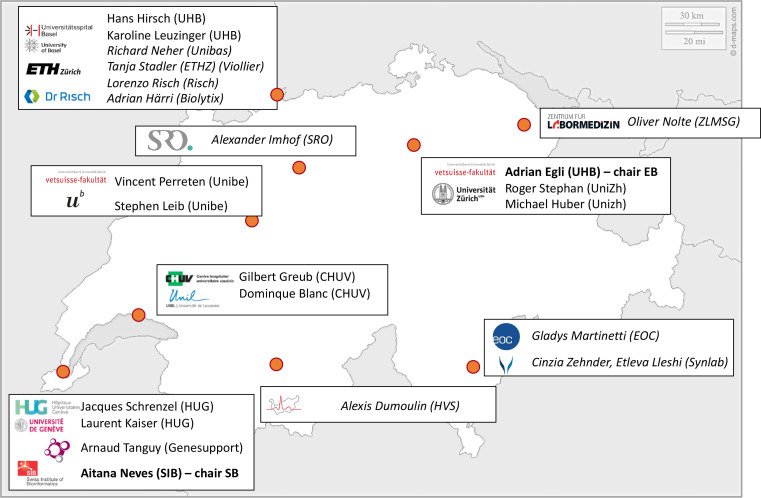
Map of institutions contributing data to SPSP. These partners have a data sharing agreement with SPSP. EB: executive board; SB: scientific board.

**Fig. 2.**
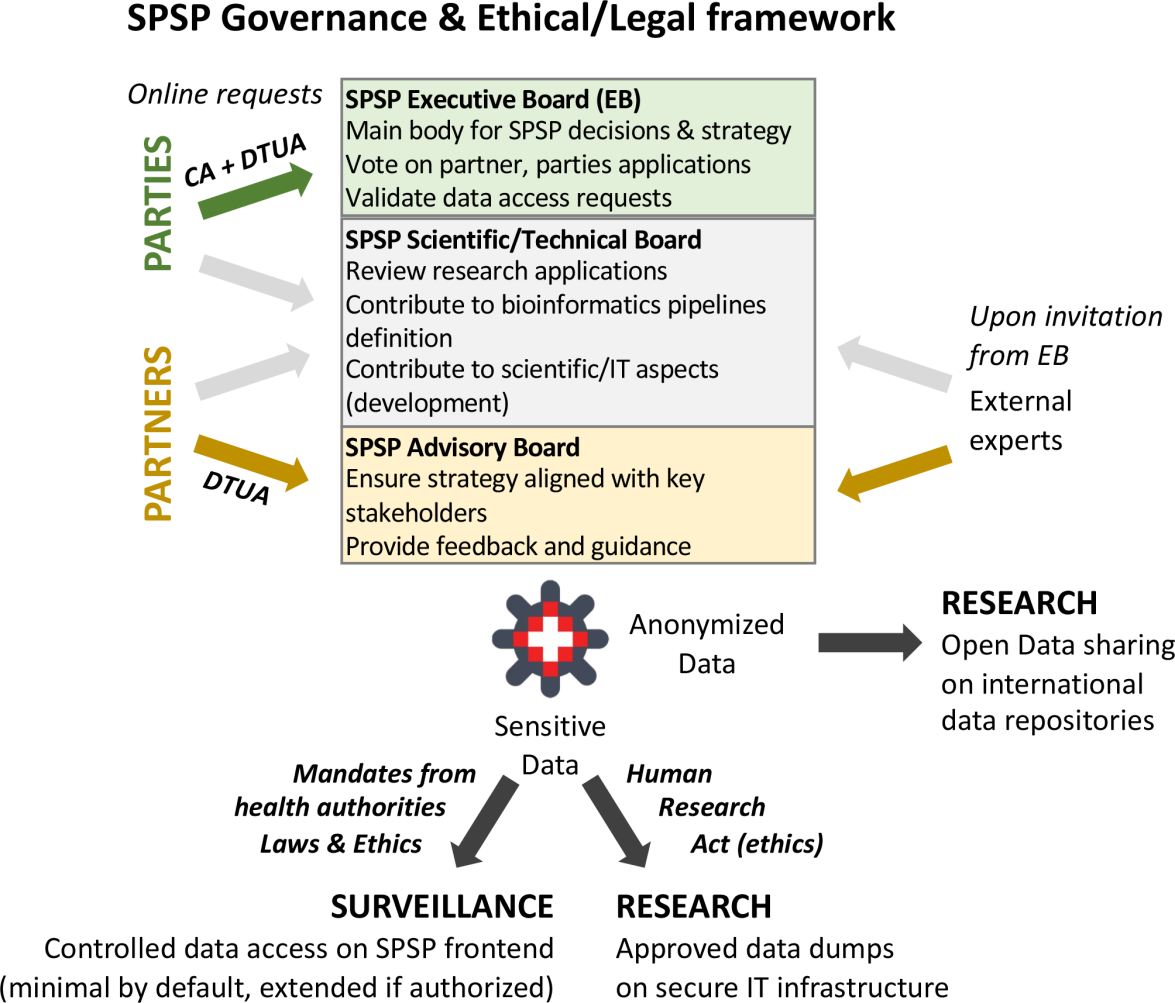
SPSP governance and ethical/legal framework. Representatives of the PARTIES constitute the Executive Board, which is the main decision body of SPSP. Upon request using the SPSP online form, new groups can ask to join SPSP as PARTIES (if interest in multiple pathogens) or as PARTNERS (if interest in a specific pathogen or project). PARTNERS are invited to join the Advisory Board. Experts from various institutions are invited to the Scientific Board. Access to sensitive data is carefully controlled with limited access to only minimal, non-sensitive data, by default, which can be extended to sensitive data upon acceptance by authorized bodies (ethics commissions, health authorities). Sensitive data may only be shared within secure IT infrastructures. Anonymized data are shared on public repositories to foster Open Data. CA: Consortium Agreement: DTUA: Data Transfer and Use Agreement.

The SPSP consortium is rooted on a well-defined legal, ethical and governance framework. In addition to the five founding Parties, ten diagnostic and research laboratories currently support the SPSP consortium as project partners and thus contribute significantly to the surveillance and risk assessment of pathogen evolution in Switzerland (Fig. 1). The consortium agreement (CA) and data transfer and use agreement (DTUA) have been developed based on the Swiss Personalized Health Network (SPHN) incentive (www.sphn.ch) with a publication guideline based on the Swiss human immunodeficiency virus (HIV) cohort study (www.SHCS.ch). Importantly, all signatories abide by SPSP data being shared to international repositories in an open data philosophy where possible, while sensitive data are treated according to FAIR principles (findable, accessible, interoperable and reusable) [14]. This contractual work was approved by the legal services of all participating institutions and signed in early 2021. The legal framework also defines the governance, which comprises an executive board, a scientific board and an advisory board (Fig. 2). The executive board is chaired by a single person over a 4 year term and consists of one representative from each member institution (party) with a voting right mainly for strategic decisions. Proposals are accepted by majority vote. The broader scientific board consists of scientists and bioinformatics experts who address the technical aspects of SPSP. The advisory board consists of various stakeholders such as the FOPH, FSVO, SwissNoso (www.swissnoso.ch), anresis (www.anresis.ch), and various additional public health and research institutions. The platform was ethically evaluated and approved by all respective ethical committees in Switzerland (Nr. 2019–01291) and the study has been registered at clinicaltrials.gov (NCT04172025).

### Controlled data access and secure IT at its core

**Fig. 3.**
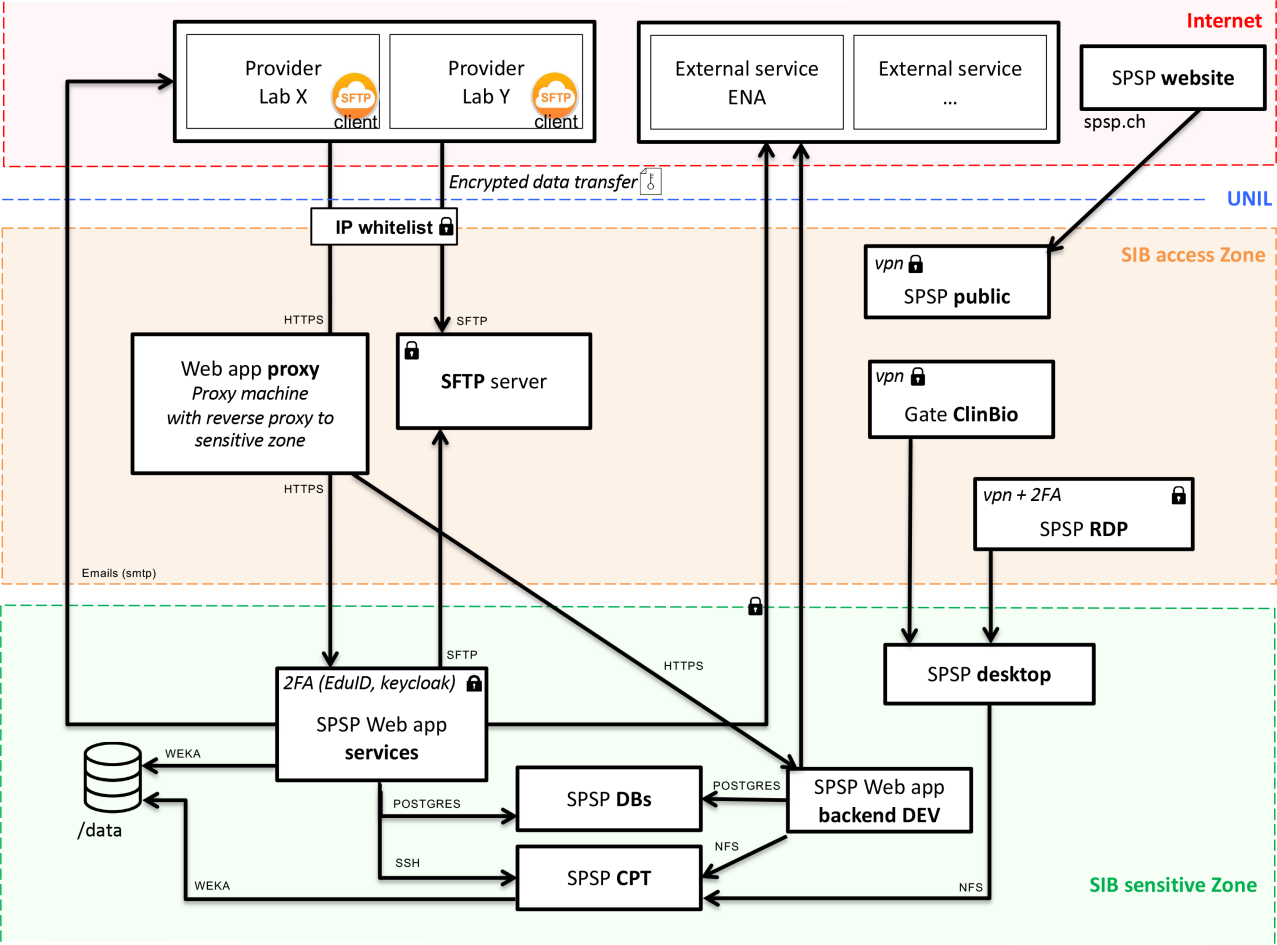
SPSP architecture. SPSP is hosted on the SIB sensitive zone, which is the Lausanne BioMedIT node. The Vue.js web application is only accessible with two-factor authentication (managed with keycloak and SWITCH edu-ID, a secure Swiss digital identity) and for white-listed IP addresses. It communicates through dedicated APIs (application programming interface) with the Java backend that has access to the databases (DBs) and compute cluster (CPT). Developers and data curators can access the sensitive zone through a secure proxy server. Data are submitted to SPSP via the SPSP SFTP server, using the SPSP Transfer tool that encapsulates, compresses and GNU Privacy Guard (GPG)-encrypts every batch of data. Authentication on the SFTP server is managed by Secure SHell (ssh) keys and access is also controlled by IP whitelisting.

SPSP enables near real-time sharing of microbial genomic data and associated metadata under controlled access. By default, users can only access a minimal subset of metadata fields on all samples. Extended access may be granted on additional sensitive metadata if authorized by the competent authorities (e.g. ethical commission for a research study) and the Executive Board. The SPSP online portal is managed by SIB (Swiss Institute of Bioinformatics) and hosted on the BioMedIT infrastructure developed within the SPHN initiative [15], which ensures high data security standards [16] (ISO/IEC 27001 : 2013, ISO/IEC 27002 : 2013; https://sphn.ch/document/information-security-policy/) and is the Swiss public infrastructure for hosting sensitive biomedical data for research use. The SPSP IT architecture is separated into an SIB access zone and the SIB sensitive zone on BioMedIT (Fig. 3). All data transfers are encrypted and access to the web portal requires IP-whitelisting and two-factor authentication. SPSP currently has a capacity of 18 Tb and this may be expanded as needed.

### Structured, curated data and harmonized pipelines for interoperability

**Fig. 4.**
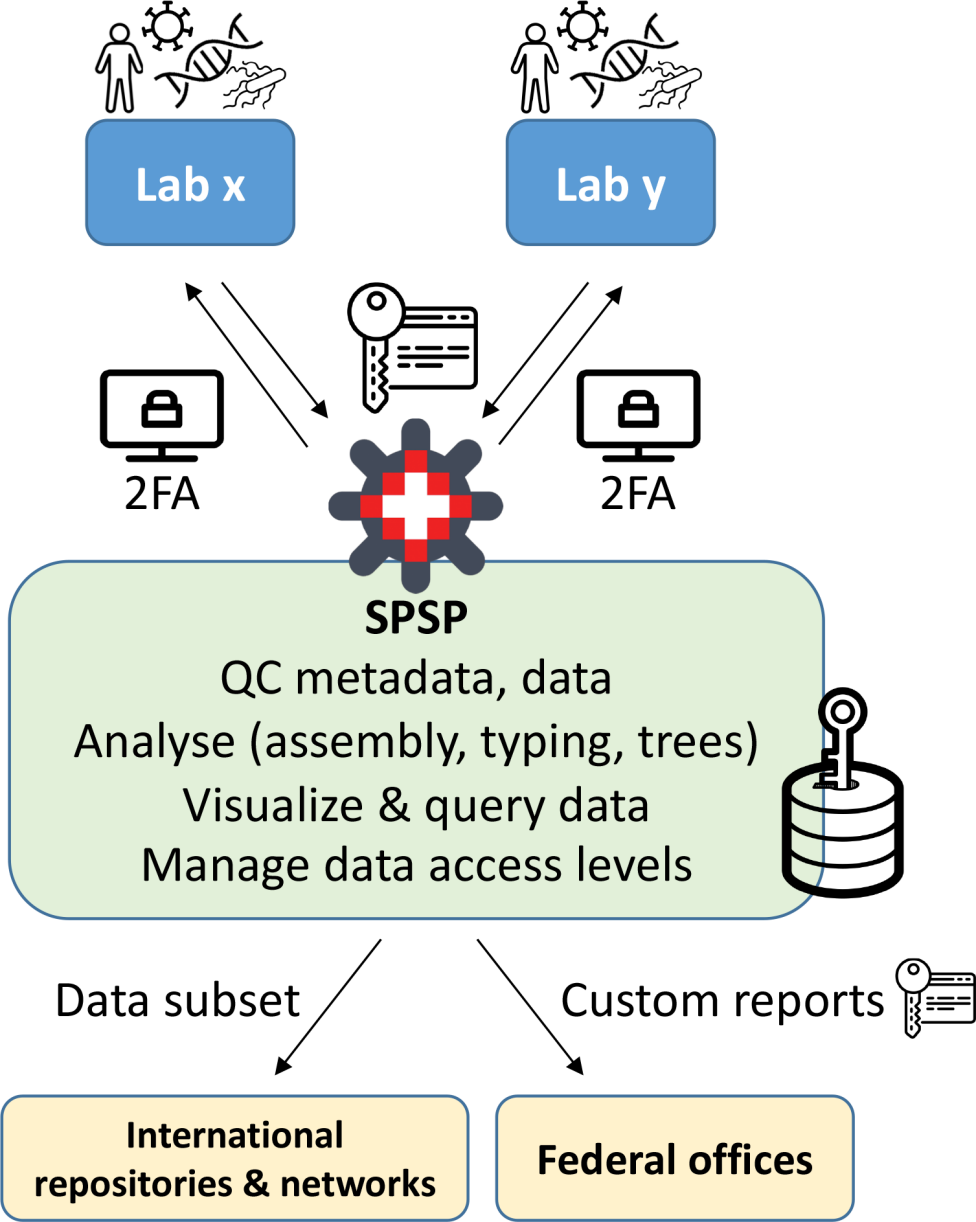
SPSP is a secure online platform for the analysis and sharing of One Health epidemiological and molecular data. 2FA: two-factor authentication. All icons from NounProject.com.

SPSP acts as a central data repository securely collecting, hosting and analysing sequencing data and their associated metadata. Incoming data are quality-checked, curated, standardized where needed and processed/annotated with dedicated bioinformatics pipelines to finally be transmitted to other databases (Fig. 4). This process has been successfully applied during the SARS-CoV-2 pandemic with the processing of ~3000 samples per week during the peaks. Additional pathogens will be handled in a similar way.

First, clinical laboratories prepare sample data including raw genomic data, consensus genome data and associated clinical/epidemiological metadata using the SPSP metadata template file, which ensures minimum interoperable metadata for a surveillance database. This also includes a data update mechanism. SPSP uses controlled vocabularies and ontologies where possible (e.g. SNOMED-CT [17], NCBI Taxonomy [18] and ATC for drugs (planned) [19]). The antibiotic resistance vocabulary follows the guidelines of the European Committee on Antimicrobial Susceptibility Testing (EUCAST, www.eucast.org) and guidelines of the Clinical and Laboratory Standards Institute (CLSI, www.clsi.org).

Second, data transfer is performed with the ‘SPSP transfer tool’, which ensures an encrypted data transfer using IP whitelisting, and ssh authentication. The data and metadata transferred to SPSP are quality controlled and loaded into the SPSP database.

Third, dedicated standardized analysis workflows are run on the data to prevent dissemination of sensitive data (removal of human sequences), for quality control and subsequent curation and annotation (e.g. for SARS-CoV-2: lineage calling using pangolin [20], variant calling and phylogenetic trees with nextclade [21]). Bioinformatics pipelines are now being developed in a modular fashion using nextflow DSL2 [22] and are published on Gitlab to foster contributions from the community (currently in development; https://gitlab.sib.swiss/clinbio/spsp-ng/spsp-ng-bioinformatics). We rely as much as possible on nf-core modules. The subworkflows will cover sequence quality checks, assembly, typing, variant calling, comparative analyses (phylogenies), as well as antimicrobial resistance and virulence predictions.

Fourth, subsets of the data are then shared with international databases (anonymized) or federal offices (secure transfers). Finally, each registered user can access the data on the SPSP frontend, query it and visualize dashboards. The data are also planned to be mapped to the Resource Description Framework (rdf) format [23] to comply with standards introduced by the SPHN incentive and to connect healthcare-related data resources across the country. Due to these security and organizational standards, SPSP is allowed to host sensitive information, which enables public health authorities to cross-link data received from SPSP with additional data sources, such as the medical or laboratory notification forms of specific infectious diseases. This allows the integration of sequencing data with additional clinical and epidemiological data.

### Serving both research and surveillance: the SARS-CoV-2 use case

SPSP serves two main objectives, providing data infrastructure for both pathogen surveillance and research. To date, successful and rich experience has been made when dealing with SARS-CoV-2 data during the pandemic, as further explained below. We are also currently developing a series of non-SARS-CoV-2-related setups including various bacterial pathogens such as *Legionella pneumophila *, *Mycobacterium tuberculosis *, *Capnocytophaga canimorsus *, *Campylobacter coli/jejuni* and many more (+10 000 datasets collected so far including, notably, *Campylobacter* spp.*, Clostridioides difficile*, *Enterococcus* spp., *Echerichia coli*, *Klebsiella* spp., *Legionella pneumophila *, *Mycobacterium tuberculosis *, *Pseudomonas aeruginosa *, *Staphylococcus aureus *, *Corynebacterium diphtheriae,* among others).

**Fig. 5.**
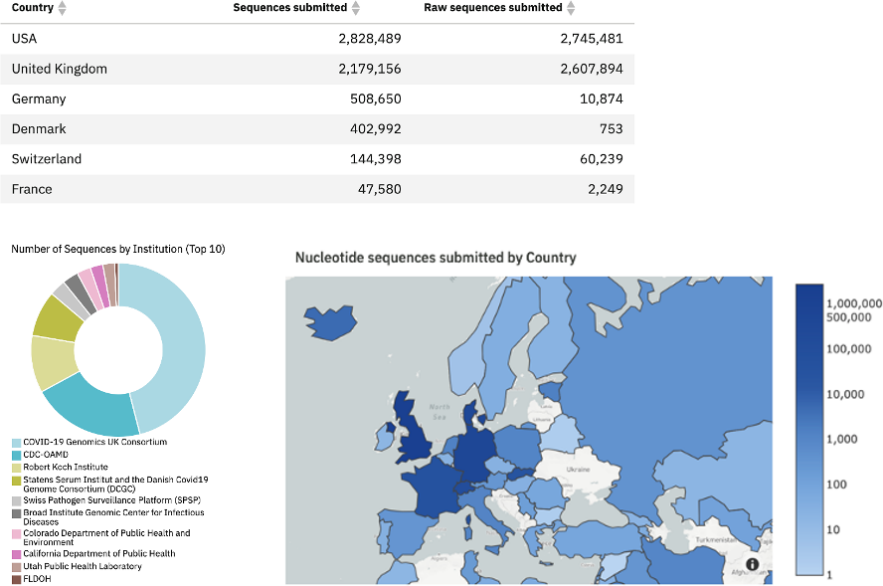
Switzerland’s contribution to global SARS-CoV-2 surveillance open data (top). Contribution by institution (bottom left). Contribution by country (bottom right). Graphs and table from COVID-19 Data Portal, as of October 2022, available at https://www.covid19dataportal.org/ and described in Harrison *et al*. [9].

During the actual pandemic, the WHO and other public health agencies recommended the use of WGS for variant tracking and identification (https://www.who.int/activities/tracking-SARS-CoV-2-variants). Therefore, at the beginning of 2021, SPSP was mandated to centralize and share Swiss sequencing data with GISAID and public repositories such as the European Covid-19 Data Portal. Switzerland and SPSP thereby strongly contributed to the international effort to monitor and fight against pandemics [24]. SPSP received sequence data from two sources: (a) the national surveillance programme financed by the FOPH and coordinated with the national reference laboratory on emerging viruses (CRIVE) at the University Hospital Geneva – in this programme, several laboratories submitted up to 3000 sequences per week from randomly selected samples; and (b) diagnostic sequencing, such as in the case of breakthrough infection or reinfection after vaccination, or suspected local outbreaks, as requested by hospital and cantonal physicians. As of October 2022, SPSP had collected and annotated more than 140 000 SARS-CoV-2 sequences, making Switzerland one of the top five contributors to global SARS-CoV-2 surveillance open data on the EU Covid-19 Data Portal (Fig. 5). SPSP also automatically shared reports on circulating variants (lineages) and mutations with FOPH three times per week, which were then published on the national Covid-19 dashboard (https://www.covid19.admin.ch). In addition, thanks to the fact that SPSP collected sensitive data on the sample primary identifiers, the FOPH was able to link each sequence with additional epidemiological data that they received from the laboratory and medical compulsory notification forms, ensuring that SPSP data were not siloed. Over 2023, it is planned that the sequencing of SARS-CoV-2 will continue in Switzerland with about 500 sequences per week, as decided by the FOPH.

### Data visualization and exploration

**Fig. 6.**
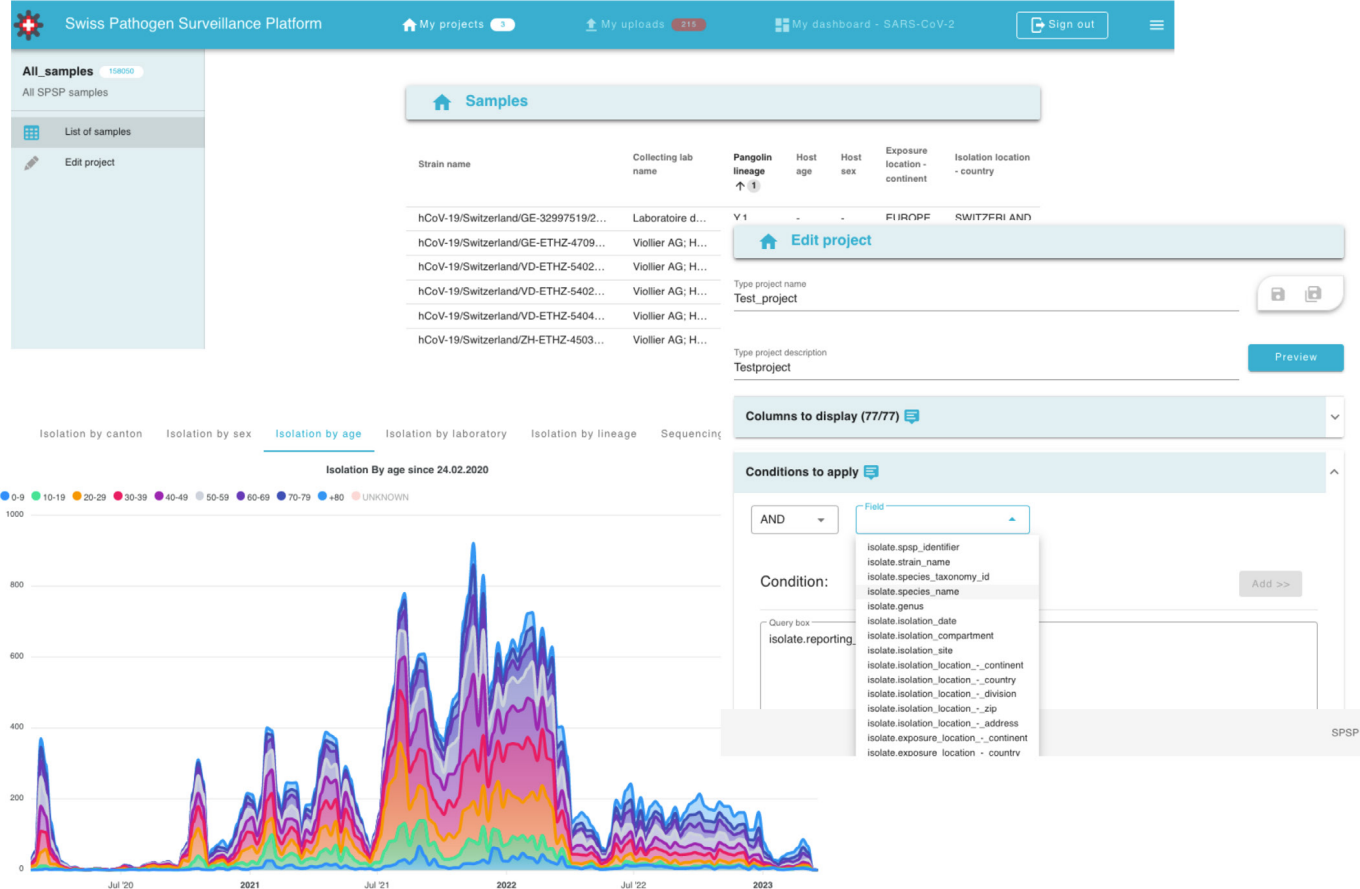
Frontend screenshots of SPSP. Upper-left: project view with four samples and six fields (columns). Middle-right: creation of a new project with the query building tool. Bottom-left: plot from the SARS-CoV-2 dashboard illustrating submissions stratified by age category of patients.

A frontend to SPSP was developed to allow registered users to actively monitor and explore the data uploaded to SPSP. By default, users have access to minimal data on all the samples submitted to SPSP by any user. Data exploration is enabled with the creation of dynamic, self-updating, projects that are based on sophisticated queries defined by the user. This enables searching but also monitoring, in near real-time, isolates harbouring a mutation of interest, as they are being sequenced and uploaded to SPSP by all associated centres. For each project, the user can select which fields (to which they have access) should be displayed on the browser by default, to avoid cumbersome large tables. Users can also track their data uploads via the SFTP server and follow-up any errors or missing files reported during data upload to the SPSP database. Lastly, users can also explore data with dashboards that are specific to a pathogen (e.g. SARS-CoV-2) or more general [e.g. antimicrobial resistance (not yet developed)]. Plots contain aggregated data from all submitting laboratories, but also laboratory-specific data to enable, for example, quality assessments. For SARS-CoV-2, the dashboard consists of nine different views showing notably submissions stratified by various criteria (patient age categories, canton, etc.), lineages over time, data quality over time using several metrics (notably from nextclade [21]) and turnaround times over time (Fig. 6).

### Contributing to international best practices

Through the ELIXIR CONVERGE initiative (https://elixir-europe.org/about-us/how-funded/eu-projects/converge/wp9), SPSP is part of a European network of SARS-CoV-2 regional/national genomic data hubs that regularly discuss best practices in terms of data standards, open data sharing and evaluation of a resource’s maturity. This ensures that current and future developments are in line with international guidelines and best practices.

## Conclusion

SPSP represents a crucial platform for the molecular monitoring of microorganisms occurring in humans, animals and the environment, enabling a One Health approach at a national level. After passing the maturity test during the COVID-19 pandemic, it is now time to expand the platform to other pathogens to monitor multi-drug-resistant and hypervirulent bacteria from humans, animals, the environment and food, as well as other respiratory viruses such as influenza. For each new pathogen, the SPSP data model will be expanded to account for pathogen-specific metadata and a dedicated dashboard will be implemented. We are also currently expanding SPSP to integrate other data formats and associated analytical pipelines, planning soon MALDI-TOF, metagenomics, and long-read sequencing data. In the longer term, we envisage that SPSP could be used for the surveillance of all pathogens of compulsory notification, generating rich data that could also serve research and outbreak investigations. Key challenges remain to secure sustainable funding for SPSP from, for example, public health agencies, and to further interface SPSP to other platforms internationally such as the EFSA, as an essential step to provide a comprehensive ‘One Health’ surveillance and outbreak monitoring system for infectious diseases beyond COVID-19.
